# How underappreciated autoinflammatory (innate immunity) mechanisms dominate disparate autoimmune disorders

**DOI:** 10.3389/fimmu.2024.1439371

**Published:** 2024-09-20

**Authors:** Kerem Abacar, Tom Macleod, Haner Direskeneli, Dennis McGonagle

**Affiliations:** ^1^ Department of Internal Medicine, Division of Rheumatology, Marmara University School of Medicine, Istanbul, Türkiye; ^2^ Leeds Institute of Rheumatic and Musculoskeletal Medicine, University of Leeds, Leeds, United Kingdom; ^3^ National Institute for Health Research, Leeds Biomedical Research Centre, Leeds Teaching Hospitals, Leeds, United Kingdom

**Keywords:** autoimmunity, autoinflammatory diseases, neutrophil, MHC-I antigen presentation, autoantibodies, immune cells

## Abstract

Historically inflammation against self was considered autoimmune which stems back to the seminal observations by Ehrlich who described serum factors, now known to be autoantibodies produced by B lineage cells that mediate “horror autotoxicus”. The 20^th^ century elucidation of B- and T-cell adaptive immune responses cemented the understanding of the key role of adaptive immune responses in mediating pathology against self. However, Mechnikov shared the Nobel Prize for the discovery of phagocytosis, the most rudimentary aspect of innate immunity. Fast forward some 100 years and an immunogenetic understanding of innate immunity led to the categorising of innate immunopathology under the umbrella term ‘auto inflammation’ and terminology such as “horror autoinflammaticus” to highlight the schism from the classical adaptive immune understanding of autoimmunity. These concepts lead to calls for a two-tiered classification of inflammation against self, but just as innate and adaptive immunity are functionally integrated, so is immunopathology in many settings and the concept of an autoimmune to autoinflammation continuum emerged with overlaps between both. Herein we describe several historically designated disorders of adaptive immunity where innate immunity is key, including rheumatoid arthritis (RA), systemic lupus erythematosus (SLE), systemic juvenile idiopathic arthritis (sJIA) and adult-onset Still's disease (AOSD) where the immunopathology phenotype is strongly linked to major histocompatibility complex (MHC) class II associations and responds to drugs that target T-cells. We also consider MHC-I-opathies including psoriasis and Behcet's disease(BD) that are increasingly viewed as archetype CD8 T-cell related disorders. We also briefly review the key role of barrier dysfunction in eczema and ulcerative colitis (UC) where innate tissue permeability barrier dysfunction and microbial dysbiosis contributes to prominent adaptive immune pathological mechanisms. We also highlight the emerging roles of intermediate populations of lymphocytes including gamma delta (γδ) and mucosal-associated invariant T (MAIT) cells that represent a blend of adaptive immune plasticity and innate immune rapid responders that may also determine site specific patterns of inflammation.

## Introduction

Inflammation represents both a repair and a defence mechanism against perceived threats to self (1). More than a century has passed since Paul Ehrlich, the 1908 Nobel laureate, posited that inflammation mistakenly directed against self would trigger what he termed “Horror Autotoxicus.” With the discovery of autoantibodies, the concept of autoimmunity emerged including a multitude of mechanisms driving immunopathology (2).

Commencing in the second half of the 20^th^ century, the genetic architecture of the murine major histocompatibility complex (MHC) that underpinned transplant rejection and immunity began to emerge (3). A near identical model was discovered in humans that were termed as human leukocyte antigens (HLA) (4). Subsequently, the classification of HLA molecules into class I and class II and the relationships between T- and B-cells and MHC peptide presentation, a compelling basis for adaptive immune system, cellular and humoral immunity emerged along with abnormal lymphocytic reactions to self-antigens that firmly underpinned autoimmunity (5, 6). Such is the veracity of the autoimmunity concept that many diseases can be well controlled by specific targeting of B- or T-cells. The veracity of the concept also resulted in immunological overreach with the attribution of virtually all non-infectious inflammatory pathologies as autoimmune.

Mechnikov shared the Nobel Prize with Ehrlich for the discovery of phagocytosis, one of the basic functions of innate immunity (7). About a century later, the autoinflammation concept emerged with the identification of ectodomain mutations in the p55 tumour necrosis factor (TNF) receptor in patients with fever and diffuse inflammation (TNF-receptor-associated periodic syndrome, TRAPS) where discernible autoimmunity was lacking (8). This discovery followed fast on the heels of the demonstration that familial Mediterranean fever (FMF), a monogenic disease characterised by excessive neutrophilic inflammatory responses, was due to gain of function mutations in the *MEFV* (Mediterranean fever) gene which encodes pyrin, a critical activator of the inflammasome expressed in myeloid lineage cells (9, 10). This emergent understanding of innate immunity led to the categorising of innate immunopathology under the umbrella term ‘autoinflammation’ and terminology such as “horror autoinflammaticus” to highlight distinctions from the classical adaptive immune understanding of autoimmunity (11). Self-directed inflammatory pathologies in which local factors in disease-specific sites lead to the activation of innate immune cells and cause target tissue damage, such as impaired homeostasis of canonical cytokine cascades (as in periodic fevers) or abnormal bacterial perception (as in Crohn’s disease), were defined within the scope of autoinflammatory disease (2).

Although classified separately, the innate and adaptive immune systems are functionally integrated and in constant interaction with each other (**Table 1**). When discussing innate immunity as a driver for disease there are two key considerations - the innate immune cells themselves that reside in or circulate to the target tissue and the target tissue non-immune compartment. For example, barrier disruption in the skin compartment best characterised by filaggrin single nucleotide polymorphisms (SNP) leading to disruption of barrier integrity allows antigenic access that ultimately culminates in T-helper (Th) 2 immune responses and eczema or atopic dermatitis (AD) (12). Although we conceptualise the therapy of AD through immunomodulation, for example with interleukin (IL) 4/13 or Janus Kinase (JAK) inhibition, it is fascinating that children with AD can be treated with “wet wraps” that at the simplest level restore epidermal barrier homeostasis (13). Herein, we further describe these complex interactions in rheumatic disease settings, where the integration of both the innate and adaptive immune system work in unison to determine phenotypes that are too often pigeonholed into either autoimmunity or autoinflammation where in reality there is functionally integrated immunopathology.

**Table 1:** Links between innate and adaptive immunity.

Component	Several Functions	Prototype Diseases
**Complement**	• C1q attenuates the effect of ICs to increase IFNα expression. • C3b, in combination with ICs, bind to and activate T cells. • C4 provides negative selection of autoreactive B cells.	Systemic Lupus Erythematosus
**Fc Receptors**	• Fc receptors on cells such as monocytes and macrophages interact with antibodies to initiate a series of immune responses.	Systemic Lupus Erythematosus, IgA vasculitis, Ulcerative colitis,
**Innate lymphocytes**	• Rapid cytokine production independent of peptides.	Spondyloarthritis, Rheumatoid Arthritis
**Neutrophils**	• Neutrophils migrate to the site of inflammation by IL-17 and augment inflammation.	Spondyloarthritis, Behcet’s Disease
	• Self-nucleic acid released by NET formation acts as autoantigen.	Systemic Lupus Erythematosus, ANCA associated vasculitis
**Macrophages**	• Increases antigen presentation from HLA-DRB1*0401 by increasing citrullination by Peptidyl Arginine Deiminase 4.	Rheumatoid Arthritis
**IL-17**	• IL-17 released from lymphocytes ensures the migration of neutrophils to the inflammation site.	Spondyloarthritis, Behcet’s Disease
**IL-18**	• IL18 can be produced in high amounts by hepatic macrophages and can trigger the Th1 response that causes IFNγ secretion by cytotoxic CD8 lymphocytes.	adult onset Still's disease
**Type I Interferons**	• Type I Interferons increase autoantibody release from B cells both directly and by increasing Blyss release from monocytes.	Systemic Lupus Erythematosus

(IFN, Interferon; IC, Immune complexes; Fc, Fragment crystallizable; Ig, Immunoglobulin; IL, Interleukin; NET, Neutrophile extracellular traps; HLA, Human leukocyte antigen; Th, T-helper).

## Rheumatoid arthritis

It is now well established that both human and murine experimental immunopathology is heterogeneous and clinical disease phenotypes may be predominantly autoimmune or predominantly innate immune (14). Rheumatoid arthritis (RA), for example, has shared epitope positive, rheumatoid factor (RF) and anti-citrullinated protein antibody (ACPA) positive cases that respond to B- or T-cell targeting with agents such as Rituximab or Abatacept which are very much in the autoimmune spectrum (15, 16). However, it is also clear from monogenic disorders including FMF and Blau syndrome which develop arthritis and synovitis that closely resemble that of RA, that an RA phenotype can be exclusively innate immune mediated with neutrophilic or macrophage driven inflammation and responds to therapies including colchicine or anti-cytokine blockers (17). Nonetheless, in RA the autoantibodies are associated with immune complex formation that is thought to engage macrophages, inducing the production of cytokines including tumour necrosis factor (TNF) in particular. Indeed, this may underscore the seminal translational immunology studies that transformed disease when TNFα blockade was found to be effective (18) and further demonstrates the effectiveness of both innate and adaptive therapies in RA (19). Another fascinating feature of RA is the innate immune barrier dysregulation in the gingiva and in the lung with non-specific citrullination that paves the way for the later autoimmunity in the joint (20). Thus, going forward, it may be possible to envisage targeting innate immunity to prevent ACPA + RA and then adaptive immunity for clinically established disease. An obvious iteration of this is to block non-specific macrophage mediated innate immune citrullination of peptides to target disease.

**Figure 1:**
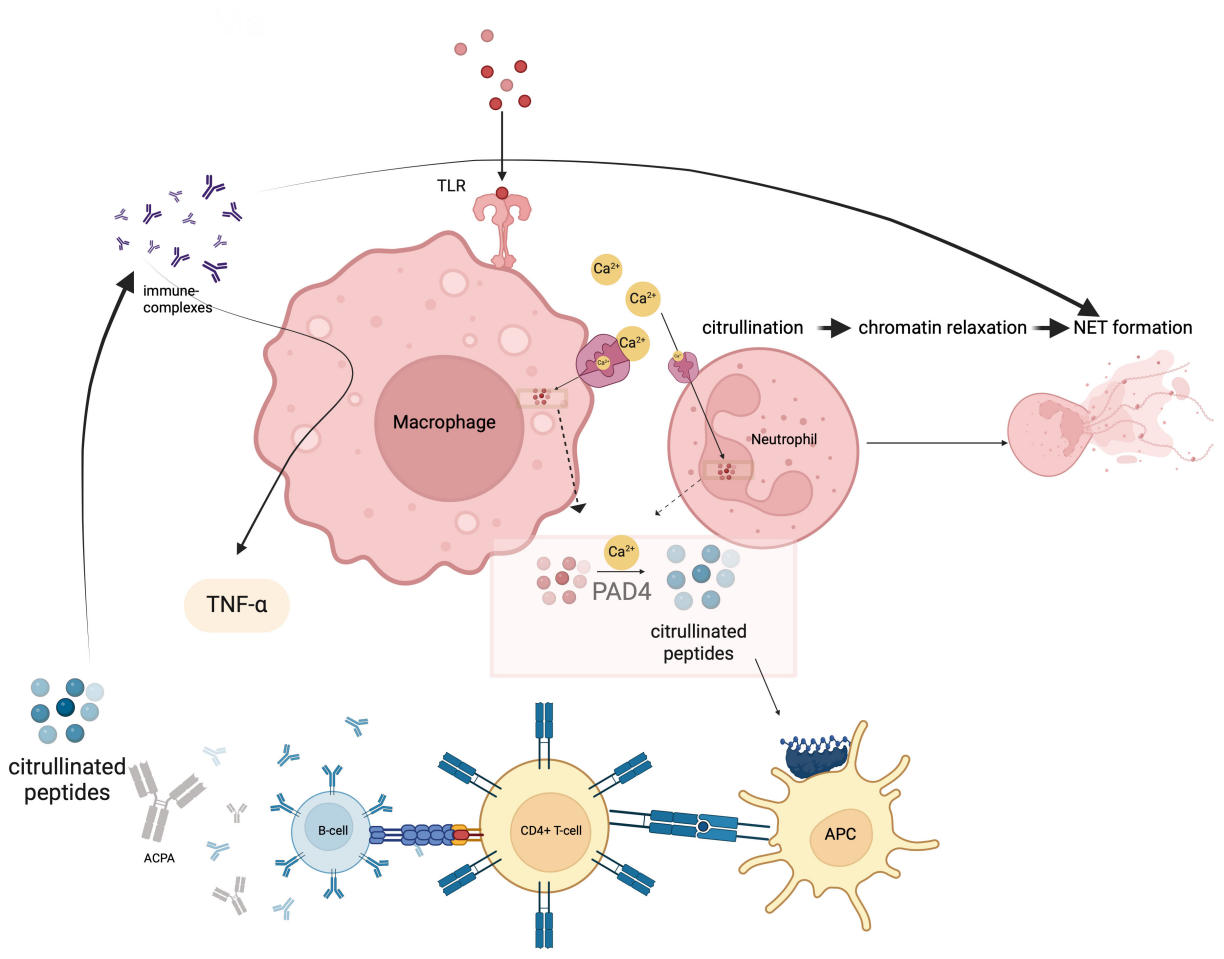
Citrullination occurs via the enzyme peptidyl arginine deiminase-4 (PADI4) and likely predates autoimmunity and occurs at sites of non-specific inflammation such as the gums and lungs where macrophages and neutrophils contribute to such citrullination processes. Afterwards, this innate immune phase leads citrullinated peptides presentation to CD4 Tmphocytes via MHC-II peptide presentation. ACPA production is induced after T and B lymphocyte interactis With citrullinated proteins immune complex formation and FcR binding on macrophages, TNF and other cytokine release occurs. In effect autoimmunity is sandwiched between Innate immune Initiation events and innate immunity terminal effector events. This is likely why RA does not cross the placenta and mediated neonatal autoimmunity.

RA presents a wide range of classification patterns including clinical and pathogenetically different mechanisms and manifestations. Although these all fall under the umbrella of RA, it is obvious that they are different entities (17). Based on the interaction of B-and T-cells, MHC-II associated ACPA-driven disease is a picture that meets the classical definition of autoimmune RA with its unique response to B-cell targeted therapies (21). Antigen presentation of citrullinated proteins produced by endogenous or exogenous factors that may be ACPA targets occurs through MHC-II related mechanisms, and this constitutes one of the most important steps in going from autoimmunity to a frank autoimmune arthritis (22). Citrullination is a post-translational modification process in which arginine in peptides are transformed to citrulline via “peptidyl arginine deiminase” (PAD) (23). The antigenic potency of arginine residues enzymatically modified to citrulline by PAD, a myeloid cell derived enzyme at sites of inflammation, underscores the key early autoinflammatory component. In this context, macrophages, monocytes and neutrophils make an important contribution to this classic autoimmunity of RA with their key role in PAD4 production (24). Indeed, calcium flux leading to increased cytosolic Ca^2+^, an event that occurs during macrophage activation, NETosis in neutrophils and during apoptosis, activates PADs by inducing essential conformational changes after Ca^2+^ binding (25, 26). The reason for such increased antigenic effects is the difference in binding potential of citrullinated peptides to MHC peptides in antigen presenting cells (APCs). In particular, the interaction of citrulline vimentin with the P4 pocket of HLA-DRB1*04:01/04, which is specific for RA and called the shared epitope, increased detection of CD4^+^ T-cells against citrullinated vimentins in the peripheral blood of RA patients and presence of plasma cells producing ACPA are the route of the classical RA autoimmunity (27, 28). In addition, exposure to some viral or bacterial agents, especially *Porphyromonas gingivalis*, triggers ACPA formation with cross-reactivity due to molecular mimicry (**Figure 1**) (29).

There are also some components of the innate immune system that upregulate PAD activity and thus increase the antigenic burden (30). Recognition of pathogen-associated molecular patterns (PAMPs) by pattern recognition receptors (PRRs), especially in macrophages, can activate PADs by inducing extracellular Ca^2+^ influx (31, 32). The production of neutrophil extracellular traps (NETs), extracellular web-like fibre network consisting of nucleic acids that can bind to microorganisms including pathogens that is an important element of the innate immune system, is also evident in RA (33). Increased neutrophil counts in the synovial fluid confirms the contribution of NETosis to the pathogenesis (34, 35). NETosis was associated with increased local PAD4 activity, whereby proteins citrullinated by PAD4 expand an autoantigen pool that fosters autoimmunity (**Figure 1**) (36–39).

In the continuation of the pathogenesis after the citrullination and formation of ACPA, there are some returns to the innate immune system components. ACPA generates immune complexes (IC) with citrullinated peptides and these ICs can stimulate TNFα secretion in macrophages via the toll-like receptor (TLR) 4/myeloid differentiation primary response (MyD) 88 pathway (40, 41). ACPA also provides M1 polarization in macrophages via interferon regulatory factor (IRF) 4 and 5 and increases their proinflammatory effects (42). In addition, ACPA, both via IC formation and alone, induces NETosis in neutrophils (35). Some the classical and critical autoimmunity of RA is sandwiched between innate immune initiation and effector mechanisms.

## Systemic lupus erythematosus

It is broadly agreed that B-cells are central to the pathogenesis of systemic lupus erythematosus (SLE) as the source of the autoantibodies responsible for the disease and SLE is the paradigmatic multi-organ autoimmune disease (43). An SLE hyperfunctioning B-cell compartment is suggested by genome-wide association studies (GWAS) in humans and functional studies in mice (44, 45). In just under two decades, it has been firmly established that innate immune pattern recognition of nucleic acids via TLR7 and TLR9 in particular is foundational in the genesis of experimental and human SLE. Such DNA and RNA bound to nuclear autoantigens appears to underpin the disease proclivity for autoantibodies against nuclear antigens (46). The pivotal role of nucleic acids in SLE phenotypes emerged from monogenic disorders due to mutations in genes linked to nucleic acid metabolism and these phenotypes are classified under the umbrella of interferonopathies (47). In the classical autoantibody-based pathogenesis cascade, the innate immune TLR pathway dysregulation serves as a bridge to autoimmunity via multifaceted type I interferons (IFN), B cell activation and disease.

The IFNs were discovered over 50 years ago by Isaacs and Lindemann and their pervasive roles in immune functions unravelled thereafter (48). Three types of interferon systems have been defined in mammals, depending on their chromosomal location, signal transduction mechanisms, and receptors: type 1, type 2 and type 3 (49). IFNα belongs to the type I IFN family and plays one of the most dominant roles in the pathogenesis of SLE (50, 51). Although IFNα is produced by many cells, the main producer in the pathogenesis of SLE was previously believed to be the plasmacytoid dendritic cell (pDC) (52). Detection of PAMPs by PRRs at the cell surface, endosomal and cytoplasmic regions initiate type I IFN production in the cell via the phosphorylation of IFN regulatory factors (IRFs) which positively regulate genes encoding type I IFNs (53).

Rather than pDCs being the key orchestrators of type 1 IFN production, it has recently emerged that the target tissues including the skin are the major producers of type 1 IFN thus pointing towards an initial innate immune barrier tissue dysregulation (54). However, clues showing that systemic inflammation is triggered in addition to skin involvement as a result of ultraviolet exposure led to the discovery of new circulating sources of type I IFN in addition to local type IFN production. Neutrophils may induce stimulator of interferon genes complex (STING)-dependent interferon signature as a result of NET formation, and also may cause neutrophil-based damage to the kidney by reverse migration, which cannot be demonstrated in pDCs (55).

**Figure 2:**
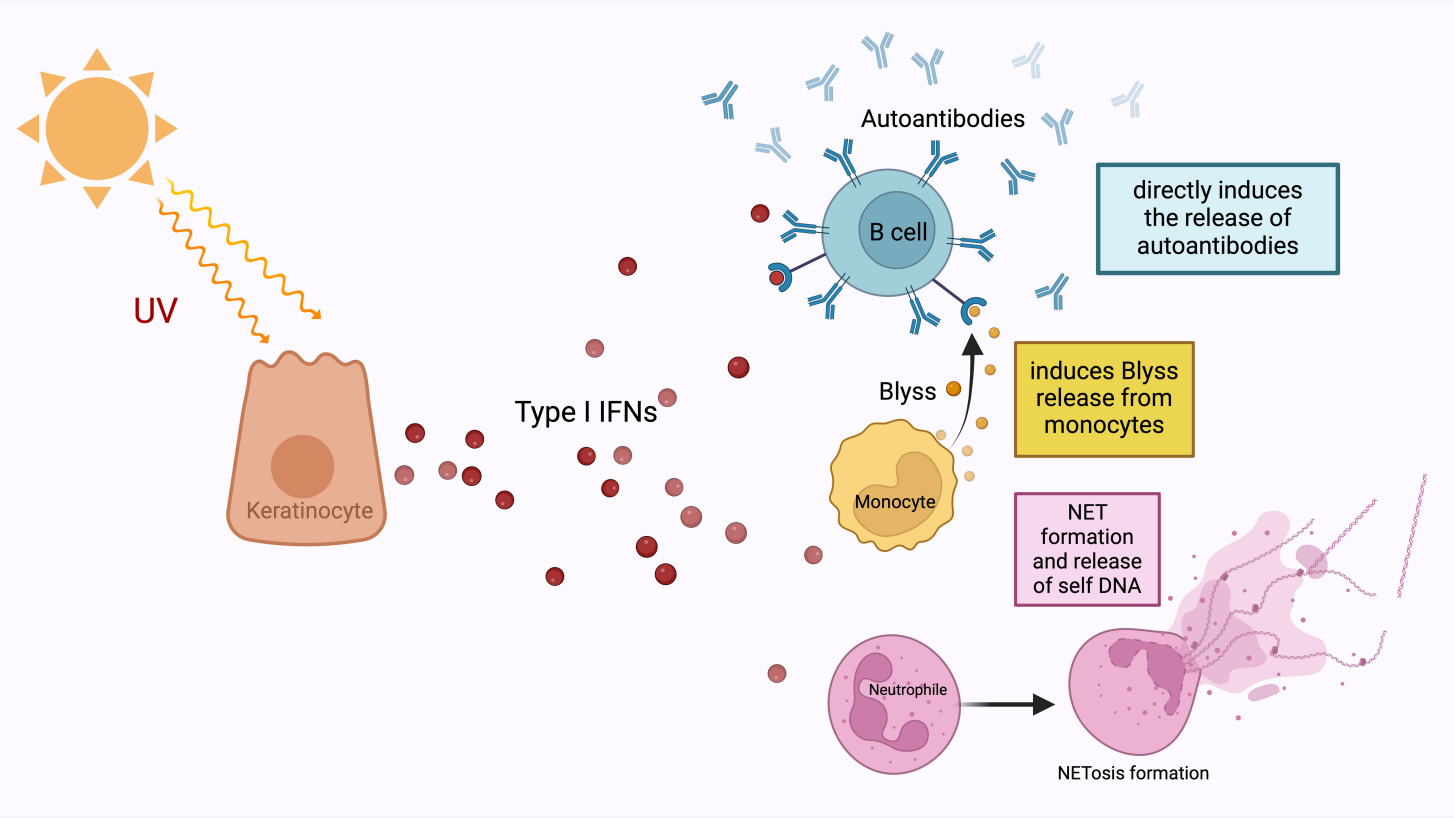
Type I IFNs are released from keratinocytes under the influence of ultraviolet light. They enable the development of autoantibodies from B-lymphocytes, which is the most important step in the pathogenesis of SLE, either directly or by stimulating the release of Blyss from monocytes. In addition to this autoantibody development, type I IFNs also increase NET formation and release nucleic acids from neutrophils. NETosis, which is one of the most important defence mechanisms of neutrophils, is an extracellular web-like fibre network consisting of nucleic acids that can bind to microorganisms including pathogens. The most important contribution of NET formation to the SLE pathogenesis is that free DNA structures that induce autoantibody formation assume the necessary autoantigen role.

Furthermore, the archetype of local IFN producers outside the hematopoietic system is IFNκ production by keratinocytes (56). This production was also detected in non-lesional skin keratinocytes (57). The key roles of type I IFNs in the process between the UV exposure of the skin and the development of the cutaneous lupus phenotype is the increase of cytokines and other chemoattractant mediators as a result of type I IFN signalling (**Figure 2**) (58). Specifically, keratinocytes increase the production of proinflammatory cytokines such as IL-6 in response to IFNκ in lupus and conversely neutralization of IFNκ suppresses their IL-6 production (59). It is still controversial whether the local production of type I IFN in keratinocytes is an inducer of the systemic involvement of the disease. However, upregulation of IFN-inducible genes in keratinocytes from non-lesional skin of lupus nephritis patients compared to keratinocytes isolated from healthy controls indicates that systemic inflammation may also be induced by innate immune system-mediated mechanisms in the skin (60). Although aforementioned studies provide important data on the potential role of keratinocytes as a source of type I IFN, the effect of local production on the diversity of the phenotypic presence of the disease is still unpredictable. Keratinocytes, as local activators of photosensitivity and cutaneous lupus involvement, appear as potential predictors and treatment targets of systemic involvement with IFN gene upregulations found in patients with systemic involvement, even if they are not exposed to sunlight. In addition, the high levels of IFNα expression in tubular epithelial cells in lupus nephritis and the evidence that astrocytes are the main source of IFNα in neuro-lupus suggest that local production may be the main driving mechanism in some clusters of the disease (61, 62).

The pathway from Type I IFNs to antibody production by B-cells via B lymphocyte stimulator (Blyss) is one of the main determinants of the transition route from the innate immune system to the adaptive immune system within the framework of SLE pathogenesis (63, 64). Type I IFNs can directly ensure the antibody production through Blyss, as well as increase the number of autoantigens, which are the raw material of the MHC system in various ways (**Figure 2**) (64). Type I IFNs reduce the clearance of apoptotic cells, which is an important source of autoantigen, via decreasing the activation of splenic marginal zone macrophages (65). They also increase NET formation and release (66). The most obvious contribution of NET formation to the pathogenesis of SLE is through providing a source of antigenic free DNA structures that induce autoantibody formation (67). NETosis is found to be associated with autoantibody levels in SLE (67). Hence the old concept of lupus as the paradigm for autoimmunity has been flipped on its head with the understanding that tissue specific IFN dysregulation is capable of driving immunopathology.

## Systemic juvenile idiopathic arthritis and adult-onset Still's disease

The connections between the innate and adaptive immune system in systemic juvenile idiopathic arthritis (sJIA) that initiate in childhood and in adult-onset Still's disease (AOSD), which is considered to be the continuation of the same disease pattern, are particularly striking in the context of macrophage activation syndrome (MAS), in which autoinflammatory hyperactivation occurs (68). AOSD exists at the crossroads of autoinflammatory and auto immune diseases because of its pathogenesis encompassing both the innate and the adaptive immune system. Although the innate immune system is the main driver of the disease through cytokines such as IL-1 and IL-6, the fact that the identified genetic susceptibility is strongly associated with the MHC-II immune system highlights the importance of the interconnections of these two systems in AOSD (69–73). HLA-DRB1*11 variants are the alleles most associated with sJIA risk (73). Again, within the MHC class II gene cluster, the incidence of AOSD was found to be more strongly associated with HLA-DR*2, DR*4, DR*7, DRB1*12 and DRB1*15 (74). MHC-II genetic predisposition reinforces the role of antigen presentation and T-cell activation in the pathogenesis (73). In addition, elevation of the biomarkers such as soluble IL-2 receptor-α subunit (also known as soluble CD25) also strongly suggest T-cell hyperactivation (75). Chimeric antigen receptor (CAR) T-cell therapy developed against specific tumour antigens triggers cytokine storm and MAS, demonstrating that gain of function of the adaptive immune system may be sufficient for the MAS phenotype. Also, the monophasic pattern of MAS developing with CAR T-cell therapy for lymphoma suggests that the phenotype might improve with the decrease in T-cell hyperactivation. It also supports the notion that an immune hypersensitivity reaction resulting from an antigen driven adaptive CD4^+^ T-cell response can trigger MAS (76, 77).

In addition, the cytotoxic function of natural killer (NK) cells was found to be impaired in patients with AOSD. After treatment, both the frequency and cytotoxic functions of NK cells improved. Some proinflammatory cytokines, especially IL-18, are thought to suppress NK cell activity. Indeed, during cytokine storms observed in MAS, NK cytotoxicity decreases (78). IL-18 was increased in the synovial tissue, serum, and lymph nodes of AOSD patient compared to healthy controls. It has also been shown that IL-18 can be produced in high amounts by hepatic macrophages and can trigger the Th1 response that causes IFNγ secretion by cytotoxic CD8^+^ T lymphocytes (79–81). Type I interferon is a key cytokine in murine models of lymphocytic choriomeningitis virus-induced MAS. In addition, these models suggest that type I interferon may be an important bridging cytokine for MAS developing in the SLE setting.

## MHC-1-opathy including Behcet’s disease

Recognising the role of tissue specific factors leading to innate immune dysregulation i.e., pathergy responses and the role of T-cells as suggested by the HLA-B*51 association we originally classified Behcet’s Disease (BD) as an intermediate between innate and adaptive immunity (2). Given the strong overlaps with other MHC-I associated conditions including the seronegative spondyloarthritis (SpA) spectrum including ankylosing spondylitis (AS), psoriasis and acute anterior uveitis (AAU), we termed these related conditions as MHC-I-opathies (82). Barrier dysfunction in the skin and inappropriate innate immune reactions at sites of mechanical stress causes secondary adaptive immune responses with neutrophilic inflammation that provoke the exacerbation and recurrence of these diseases (82).

BD and MHC-I-opathies did not fit the classic female-dominated autoimmune disease concept with autoantibodies playing a leading role but instead exhibit convergent immunology around CD8^+^ T-cells and a close link to neutrophilic inflammation (83). However, although the absolute importance of *MEFV* mutation and neutrophils was demonstrated, it is not a typical autoinflammatory disease like FMF either (84, 85). The immune pathway of BD consists of an organizational continuum, rather than a dichotomic system, especially due to the interplays, which provides the bridges between the innate and adaptive arms (86).

Neutrophils and monocytes are hyperactive in BD, suggesting an overactive innate response, most clearly observed in pathergy (skin prick) reaction. This response is dampened by microbial sterilization and decreased traumatic needle use in skin, yet exacerbated when pneumococcal antigens are given together with needle prick, clearly implicating the innate immune system (87). However, T-cell responses to local antigens such as Retinal-S antigen cross-reactive with HLA-B*27 peptides are also shown in BD patients with posterior uveitis (88). Conventional immunosuppressives such as azathioprine or cyclosporin-A are also effective in BD unlike in autoinflammatory diseases, showing an important pathological role for the adaptive immune system as well.

It is not surprising that BD is also included in the concept of MHC-I-opathies, along with SpA and psoriasis, in which significant pathogenetic schemata similarities have been identified. Barrier dysfunction in the skin, oral mucosa and inappropriate innate immune reactions at sites of mechanical stress causes secondary adaptive immune responses with neutrophilic inflammation that provoke the exacerbation and recurrence of these diseases (82). Recently, an HLA-B*51 restricted, endoplasmic reticulum aminopeptidase (ERAP)-1-Hap10 associated CD8^+^ T-cell response has been described in peptidomes generated from BD patients, similar to B*27 restricted peptidome responses in SpA patients (89).

**Figure 3:**
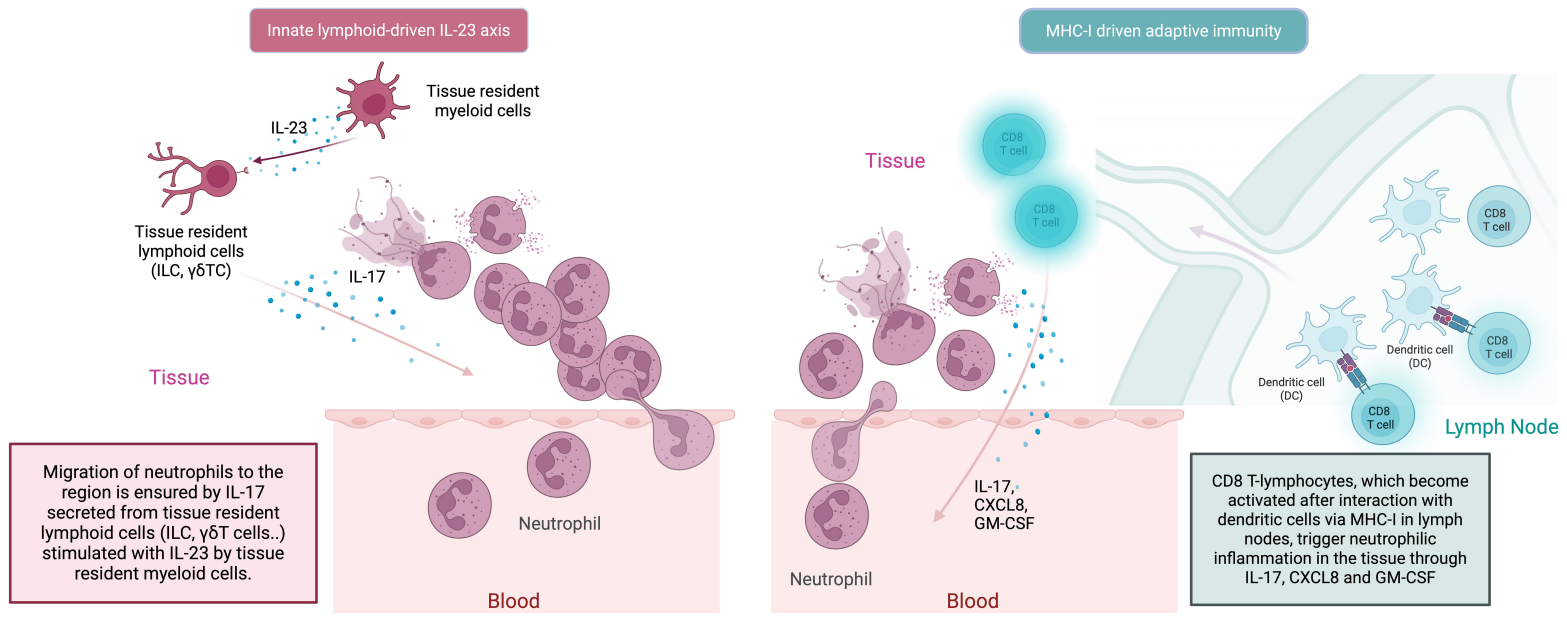
In the MHC-I-opathies, the ultimate aim of CD8 T-cell adaptive immunity is to facilitate neutrophil migration and activation at sites of Inflammation. Initially innate cells including DCs, macrophages and even neutrophils in tissues can produce IL-23. Such IL-23 release stimulates tissue resident lymphoid cells including ILC3 cells and other innate lymphocytes to produce IL-17. In parallel, APC activation of conventional T-cells in the usual MHC-1 driven adaptive pathway dramatically amplifies the release of IL-17, CXCLS and GM-CSF from CD8 T-lymphocytes to which antigen is presented by dendritic cells. This further increases neutrophil recruitment and inflammation. There the IL-23/17 axis is both a key part of both innate and adaptive immune responses at certain tissue specific sites including the skin the gut the enthesis and the uveal tract.

It is widely accepted that, as a result of the barrier perturbation that develops due to mechanical stress, the transition from the tissue-resident innate immune system to the adaptive immune system occurs via the IL-23/IL-17 axis (**Figure 3**) (82). In this cyclical process, the adaptive immune system T-cell responses are closely integrated with innate immune system neutrophilic function. Type 17 T-cells (Th17 cells, CD8^+^ T-cells, γδ T-cells, NKT-cells) play a lead role in the recruitment of neutrophils to the site both indirectly via IL-17-induced IL-8 and G-CSF (through myeloid and endothelial cells), and directly by GM-CSF production (**Figure 3**) (90). Besides Th17s, γδ T-cells and NKT-cells are also responsible for increased circulating IL-17 in BD (91, 92). In BD, as in other MHC-I-opathies, IL-17 is thought to be the main disease-driving cytokine produced by lymphocytes to stimulate the innate immune system, particularly neutrophils (93). As far as is known, the main mechanism by which adaptive immune system elements exacerbate BD is by interfering with the innate immune system and inducing peak neutrophilic inflammation. A recent novel insight relevant to BD is that neutrophils, in addition to carrying out symptom-driving tissue damage, can also produce IL-23 which offers a novel mechanism for how innate immunity could maintain the type 17 immunity (83, 94).

## Autoimmunity and barrier defects- inflammatory bowel diseases

Although the exact cause of mucosal damage is not fully known, the overarching hypothesis is that inflammatory bowel disease (IBD) pathogenesis is linked to impaired immune response against bacterial microorganisms in the intestinal mucosa in genetically predisposed individuals (95). This impairment occurs in both the innate and the adaptive immune system (96). Indeed, more than 50 monogenic forms of IBD have been described with both obvious innate and adaptive immune system mutations as drivers (97). Autoinflammatory-manner diseases due to defects in signalling pathways responsible for stabilizing the barrier and stress response, which is the foremost component of intestinal mucosal immunity, immunodeficiencies affecting granulocyte and phagocyte activation, impaired T- and B-cell selection and activation, are examples of the monogenic-based IBD phenotypes (98).

Although the barrier disruption in ulcerative colitis (UC) suggests innate immunity pre-eminence, the fact that therapy with cyclosporine, integrin blockers and S1P (Sphingosine-1 Phosphate) receptor modulators (working by blocking interaction between S1P and S1P1 receptors, which regulate lymphocyte egress from the spleen and lymph nodes into the systemic circulation, decreasing intestinal inflammation in IBD) all of which block T-cell function, attests to the major additional role played by cells of the adaptive immune system (99). However, the susceptibility loci could not explain the majority of the pathogenesis of the patients with IBD. *NOD2* (encoding nucleotide oligomerization domain receptor) and *CARD9* genes, which are especially important in innate immune system mechanisms, stand out among the defined Crohn’s disease susceptibility genes (100, 101). In addition, there are SNPs defined in *IL23R*, *JAK2*, *STAT3*, and *TYK2* genes, which encode the definitive cytokines of the IL-23/Th17 pathway cascade, and in genes that play a role in T-cell response such as *TNFSF15* (102–105).

Macrophages and dendritic cells, which both stimulate other cells of the innate immune system and present antigens to lymphocytes via MHC class II, are the main cellular bridges (106). DCs and macrophages contain innate immune receptors (pattern-recognition receptors, PRRs) such as TLRs and NOD proteins that sense pathogen-associated molecular patterns (PAMPs) of extra- and intracellular pathogens, respectively. In the adaptive immune system, triggering of antigen-specific T-cells occurs through antigen presentation by dendritic cells. The interactions of α4β7 integrin expressed on lymphocytes and mucosal address-cell adhesion molecule 1 (MAdCAM-1) in the endothelium of intestinal venules are important for the most critical step, which is the adhesion of effector and memory T-cells in the intestine (107). Hence IBD development often transitions from innate to classical adaptive immune system in disease.

IL-23, which is a vitally important link between innate and adaptive mechanisms in many of these type-17 T-cell related autoimmune diseases, is also one of the key cytokines targeted as a therapeutic agent in IBD (108). IL-23 promotes inflammation by increasing cytokines such as IL-17A, IL-17F, IL-21, IL-22, IL-26 produced from Th17 cells in the blood and intestinal mucosa (109). IL-17 produced by Th17 cells induces the secretion of other proinflammatory cytokines (e.g., IL-1β, IL-6, TNF), growth factors (G-CSF and GM-CSF) and chemokines from stromal, epithelial and myeloid cells and thus supports the migration of neutrophils and macrophages to the region (110). Although IL-6 and TGFβ must be present for naive T-cells to differentiate into Th17 cells, IL-23 also enables these cells to develop and proliferate, as well as secrete IL-17 (111).

## Autoimmunity and barrier defects- atopic Th2 diseases

Atopic diseases and most typically eczema or AD is not typically viewed as autoimmune due to the lack of autoantibodies. However, strong evidence incriminates dysregulated adaptive immune responses with Th2 lymphocytic responses via IL-4 and IL-13 mediating the cutaneous manifestations. The use of cyclosporin or anti IL-4/13 or anti-IL-13 attests to the veracity of this adaptive immunopathology (112). Yet, a key factor in the development of AD is a loss of barrier function; frequently as a result of reduced levels of filaggrin. Indeed, mutations within the filaggrin gene are the most significantly associated with AD (113). The breakdown in barrier function facilitates influx of allergens, dysbiosis in microflora which further contributes to allergen production and loss of water retention leading to dry skin and exacerbated lesions (114).

This breakdown in barrier integrity allows activation of the innate immune system and epithelial cells, causing production of Th2-driving cytokines and alarmins such as TSLP, IL-25, and IL-33, ultimately skewing the adaptive immune system to a Th2 response (115–118). Activated Th2 cells subsequently produce IL-4, IL-13 and IL-31, which directly contribute to the pathogenesis of AD by stimulating sensory neurons causing pruritis (119–121). In addition, IL-4 and IL-13 drive IgE class switching and synthesis in B cells, which is a significant driver of atopy facilitating mast cell degranulation following FcϵR binding and recognition of sensitized allergens (122, 123). Again, whilst the adaptive immune system is the crucial driver of pathology in AD following established Th2 skewing, the innate response is critical in initiating and driving Th2 development.

## Innate and non-conventional lymphocytes as autoimmunity autoinflammatory bridges

Contrary to their phenotypic and functional resemblance to T-cells, innate lymphoid cells (ILCs) do not contain acquired T-cell antigen receptors. They respond to signals from damaged tissues with the production and secretion of a range of cytokines, but do not undergo clonal selection and expansion. They have an incompletely understood relationship between the microenvironment and components of the adaptive immune response. ILCs are classified into three categories (ILC1s, ILC2s, and ILC3s) that reflect the cytokine expression profiles of classical CD4^+^ T-helper cell subsets (Th1, Th2, and Th17 respectively) (124). ILCs are stimulated by stress signals, microbial compounds, and cytokines rather than by antigens. Therefore, they tend to take an early role in immune responses. ILC1s are stimulated in response to IL-12, IL-15 and IL-18 produced by myeloid cells in response to intracellular pathogens and produce interferon-gamma. ILC2s are stimulated by epithelial-produced cytokines IL-25, IL-33, IL-4 produced from basophils, secondary to parasitic infections or allergens, leading to the production of IL-4, IL-5 and IL-13. ILC3s mainly respond to IL-1β and IL-23, which are produced by myeloid cells in response to bacterial and fungal infection. In addition, ILC3s produce IL-17 as well as GM-CSF and IL-22 during inflammation (125–132).

ILCs are not antigen-driven yet are similar to other classical T-cells in their potential for cytokine responses. They therefore provide a source of lymphocyte-derived cytokines, such as IFNγ, IL-5, and IL-13, or IL-17 and IL-22, in the local inflammation milieu early in the immune response without the need for antigen specificity. These are first responder equivalents of the classical Th1, Th2 and Th17 paradigms of adaptive immunity (133). In addition, each of these cell types reacts to distinct stimuli. ILC3s express MHC class II on their surface and are capable of presenting antigen to CD4 T-cells (134). Therefore, ILC3s regulate the activity of T-cells specific for microbiota-derived antigens through MHC-II presentation on their surface (135). Also, recently high levels of IL-17-producing ILC3s have been found in psoriatic arthritis and IBD (136, 137). In the shared pathogenesis of disease groups in the MHC-I-opathies concept such as BD, psoriasis or spondylarthritis, some tissue-located ILCs are also thought to be factors that activate the IL-23/IL-17 axis (138). Further research will be required for its other functions in systemic autoimmune diseases.

Non-conventional lymphocytes including mucosa-associated invariant T (MAIT) cells that produce IFNγ, IL-17, and IL-22, invariant NKT (iNKT) cells that produce IFNγ or IL-4, and subsets of γδ-T-cells that produce IFNγ and IL-17 have shared production of some cytokines with innate T-cells in some epithelial or mucosal tissues (139).

γδ T-cells are mostly enriched in various epithelial and intestinal tissues, as well as in the skin. Whilst non-conventional T-cells have functional yet restricted T-cell receptors (TCRs) that recognise conserved antigens, TCR signalling is not necessary for differentiation and function of non-conventional T-cells (140). Most tissue-specific γδ T-cells show a Th1, Th2, Th17, and Treg-like phenotype and are essential in inflammation (141, 142). While γδ T-cells represent a small subset of T-cells in secondary lymphoid organs, they play a unique role in the pathogenesis of IBD as they represent a major subset of intraepithelial lymphocytes (IEL) in intestinal mucosal tissues. Protective γδ T-cells perform tissue repair and epithelial cell healing during intestinal inflammation via IL-22 secretion in response to retinoic acid secreted from DCs yet can have a pathogenic effect through production of IL-17 and IFNγ. DC-induced IL-1β and IL-23 contribute to the differentiation of IL-17-producing γδ T17 cells and additionally to IL-17 and IFNγ-secreting γδ T1/17 cells (143). IL-17 induces matrix metalloproteinase (MMP) secretion by tissue resident fibroblasts, whereas IFNγ induces nitric oxide (NO) production. NO and MMPs evoke inflammation thus contribute to the development of IBD (130, 144). Powerful experimental evidence has shown how γδ T-cells are key players in experimental spondyloarthropathy that was previously thought to be more adaptive immune related or an MHC-1-opathy (145).

## Conclusions

In this article we provide translationally relevant insights into some of the key interplays between innate and adaptive immunity that manifests as autoimmune diseases. Whilst autoimmune diseases are often effectively treated by targeting B or T-cells, therapy outcomes may be variable or mixed. Numerous bi-directional links within innate and adaptive immunity could potentially create new therapeutic targets where innate immunopathology is silently smouldering in the background e.g. dysregulation of nucleic acid signalling in SLE. Moreover, each autoimmune disease has variable or even dominant innate immune drivers e.g. MEFV mutations in seronegative RA. Furthermore, environmental factors including bacteria or adjuvants could sculpt overall immune responses in different populations.

Because treatment options targeting innate immune system elements are minimal in the therapeutic armamentarium there is a need to consider adjunct strategies targeting innate immunity in autoimmune disease to more comprehensively treat autoimmunity, reduce adaptive immunity immunosuppression and target cases that are predominantly innate immune driven. The greatest success in innate immunotherapy has likely been colchicine, which target neutrophils, which we have mentioned for its active roles in many diseases. Ultimately, with the data provided by clinical and molecular studies, the autoimmune-autoinflammatory disease classifications designed hitherto will be reshaped and refined with new discoveries in the future.
